# Obstacles to recruitment in paediatric studies focusing on mental health in a physical health context: the experiences of clinical gatekeepers in an observational cohort study

**DOI:** 10.1186/s12874-019-0730-z

**Published:** 2019-04-27

**Authors:** Maria E. Loades, Lucie Smith, Nina Higson-Sweeney, Lucy Beasant, Paul Stallard, David Kessler, Esther Crawley

**Affiliations:** 1 Department of Psychology, University of Bath, Bath, BA2 7AY England, UK; 2 Bristol Medical School, University of Bristol, Bristol, UK; 3 Department of Health, University of Bath, Bath, UK

**Keywords:** Recruitment, Paediatric, CFS, Cohort study, Gatekeeping

## Abstract

**Background:**

Studies in both paediatric and psychiatric settings often experience problems in recruitment. This can compromise the ability of the study to recruit to target, meaning studies are potentially underpowered. It can also result in a biased sample if a non-representative group are selectively recruited. Recruitment to studies in health contexts often depends on healthcare professionals, who act as gatekeepers by screening patients for eligibility and obtaining consent for the research team to contact them. The experience of health professionals as gatekeepers in paediatric studies is poorly understood and may affect whether recruitment is successful or not.

**Methods:**

Six out of seven eligible healthcare professionals from a specialist paediatric chronic fatigue syndrome (CFS) team were interviewed. All participants were undertaking initial clinical assessments within which they were asked to identify eligible patients for an observational study of co-morbid mental health problems in adolescents with confirmed CFS/ME. This study had experienced particular recruitment problems, more so than other studies in the same service. Interview questions were designed to explore perceptions of research, and barriers and facilitators of recruitment. Interviews were audio recorded and transcribed verbatim. Thematic analysis was used.

**Results:**

Participants espoused their commitment to the value of research. However, they perceived there to be a number of barriers to recruitment. Barriers within the clinical context included time pressures and the emotional nature of initial clinical assessments. Barriers posed by the wider research context included recruiting to multiple studies at the same time. Factors specific to the observational study of mental health in CFS/ME included aspects of the study design, such as the name and nature of the study, as well as the focus of the study itself. Participants made a number of recommendations about how recruitment barriers could be overcome.

**Conclusions:**

The current study highlights the need to carefully consider, at design stage, how to overcome potential barriers to recruitment. Gatekeepers should be actively involved at this stage to ensure that the study is set up in such a way to best enable recruitment activities within the clinical setting.

## Background

In clinical studies, healthcare professionals often act as gatekeepers who facilitate access to potential participants by introducing the study to them and gaining their consent to share their contact details with researchers [1]. Gatekeepers also appear to influence willingness to participate; patients approached about research by their usual doctor are more likely to participate than those who were approached by someone unfamiliar to them or who saw advertisements about the research [2]. Universally, recruitment problems in the context of clinical studies are commonly experienced across settings and patient groups [3–5], including in paediatric settings [4, 6] and in psychiatric research [7–9]. Recruitment issues pose 2 problems for researchers, firstly that a study may fail to recruit sufficient participants to have the power required to detect an effect, and secondly, that the sample recruited may not be representative of the wider population to which inferences are made, affecting the validity of the conclusions drawn due to selection bias [7, 10].

A number of factors are known to influence a gatekeeper’s decision to inform a potential participant about a study. The gatekeeper needs to assess the potential participant as being eligible for the study, and then to decide to inform them about it. The health status of the patient, the gatekeeper’s engagement with the patient, and their own attitude towards research are likely to affect this decision [8]. The gatekeeper is likely to inform the patient of the study only if they perceive the potential benefits of doing so to outweigh the potential risks.

Although recruitment to studies often depends on healthcare professionals acting as gatekeepers, the views of these professionals, including barriers to recruitment and potential solutions, are poorly understood. Therefore, in the context of an observational study of mental health problems in adolescents with paediatric Chronic Fatigue Syndrome (CFS/ME) presenting to a specialist paediatric CFS team, we undertook a qualitative study to explore healthcare professionals’ views. We did this because we noticed that rates of healthcare professionals failing to undertake eligibility assessments to this study were considerably higher than for other studies in the clinic; approximately 15% of patients were not assessed for eligibility for this study as compared to < 4% in the SMILE trial [11], conducted in the same service. The aim of the current study was to further understand the barriers to recruitment to mental health studies in paediatric health populations, and to generate recommendations for how these can be addressed.

## Method

### Participants

The context was a multidisciplinary team providing specialist assessment and intervention for paediatric CFS/ME within the National Health Service (NHS) in the South West of England. This team had been recruiting for the past 20 months to an observational study screening a clinical cohort of adolescents (age 12–18 with a confirmed diagnosis of CFS/ME at initial assessment) to determine the prevalence of mental health problems. Healthcare professionals who had conducted initial assessments within the team within the prior year were eligible to participate (*N* = 7). Of these, 1 healthcare professional opted not to participate as they are also a member of the research team. Thus, 6 participants (3 medical doctors and 3 allied healthcare professionals) were interviewed. The participants were a diverse sample in terms of age, gender and years of post-qualification experience.

### Procedure and materials

Potentially eligible participants were invited to take part in an interview by email, and a participant information sheet was attached to this invitation. The study was also briefly introduced at a clinical team meeting. Interested parties were asked to contact the research team directly. Participants were asked to complete a consent form, and were subsequently interviewed via Skype (*N* = 1), over the phone (*N* = 4), or face-to-face (*N* = 1). A semi-structured topic guide exploring healthcare professionals’ views of recruiting to studies, including the facilitators and barriers to recruitment to this study specifically, was used to structure the interviews. Interviews were conducted by the principal investigator (ML), and were audio-recorded. Interviews were transcribed into Microsoft Word and anonymised at the point of transcription.

### Data analysis

Thematic analysis is a method by which patterns within a set of data can be identified and analysed [12]. We used a combination of a deductive (based on existing literature) and inductive (based on emergent data) approach. We adopted a realist perspective (i.e. that a truth exists independent of our conceptualisation of it), and identified themes at a semantic level (i.e. at the level of words and phrases). Data collection and analysis were conducted concurrently. ML conducted the interviews and subsequently read the transcripts several times as data familiarisation. ML generated initial codes and themes from the first 4 transcripts. These were reviewed and discussed with EC, DK and PS. Subsequently, this was repeated for the remaining 2 transcripts, with revisions and additions made to the initial codes by ML, which were reviewed and discussed with EC.

## Results

Three specific themes were identified: the value of research, barriers to recruitment at gatekeeping stage, and what can be done to help to overcome these barriers. Each theme (and concomitant subthemes) is summarised below, with illustrative quotes in Table 1. Individual participants are referred to by number (e.g. P1, P2, etc.).

**Table 1:** Themes and subthemes with illustrative quotes

Theme	Sub-themes		Example Quotes
Value of research	Staff are interested in and committed to research		• “it’s one of the things that really attracts me to the [location] team is because of the research links … for me as somebody who is not in a …research position and I’m not involved in the research projects, it’s really exciting for me to be involved-to have that link” (P1)
			• “it’s very positive because we’re making change to help the whole population” (P4)
			• “Every patient who comes through the door should have equal opportunity to be part of it I believe and whatever research is going on if the meet the criteria they should be given that option and it’s my role to um signpost them, signpost the information so they have the chance to be part of that.” (P4)
			• “it’s a really important role in-in-in the team as a whole and I think that, um, my job is to make sure that if there is anybody eligible that I recognise that and I offer them the opportunity to take part” (P6)
	Importance of research in developing the evidence-base		• “…I feel that gives me an added confidence with-with patients because I-I know that y’know what I’m saying is evidence-based.” (P1)
			• “… it’s so interesting really to be learning more about um people’s experiences and using that to show what we do so you know…for example, in an initial assessment when you’re talking with a young person and their family and telling them about the evidence base and the treatment you can really do so with real confidence. I mean any…clinician can generally but we can say look hey this is a study we did last year, this is what we found. I mean that’s just fantastic like live, real time use of research” (P3)
			• “Research is always valuable because we have to provide an evidence based service …and we also have to answer unanswered questions” (P5)
	Research is not seen as part of the primary role/focus of clinical staff		• “I am very pressured as a clinician, trying to hold it all in mind with things that [research] is not the emphasis of my assessment, it will not – never be-…because I’m here for clinical end of things” (P2)
			• “But um, yeah when, people -you know sort of if they’re crying...I think then you can’t really prioritise research over what’s happening” (P6)
Barriers to recruitment	Clinical context of recruitment	Time Pressure	• it can be difficult to um er because you run out of time in initial assessment to then um prioritise enough time to talk to people about the study (P1)
			• “gets pushed out from running out of time” (P3)
			• “I’ve got limited time to get all the information out.” (P4)
		Complex/emotional/demanding initial assessments	• “…how the assessment has gone and in terms of complexity and emotional load for the family” (P3)
			• “if the patient is struggling to take on information about their diagnosis let alone an additional bit of work around research. So if their mood is perhaps lower or their motivation is lower um it would be more challenging to recruit a patient like that than a sort of very proactive family that are bouncing around wanting to help and do things” (P4)
			• “you’ve got to be wary of your focus on the patient in the consultation it’s their consultation versus the demands of research which is helping the wider patient community come up with answers in the future and that’s a delicate balance you have to make and you have to judge whether it’s appropriate the moment to ask them about research or not depending on their levels of distress.” (P5)
			• “So I’m always obviously trying to think of putting myself in their shoes and thinking it’s like oh what’s – what’s – what’s good for them?” (P2)
			• I think one of the groups I’ve struggled with is when they’re really obviously struggling with their own mental health …then somehow it just seems like a difficult question to add on at the end...so, I know that’s a bias (P6).
	Wider research context	Research fatigue in the context of too many research projects	• “there is a danger of getting research overload so I think it suffered a bit because it came on the back of all the other trials we’d done and we’d just finished recruiting and people heaved a sigh of relief and said can we have a little window off recruiting please” (P5)
			• “…there were around four or five research projects, seem to be, seemingly on the go at any one time…and that is confusing.” (P2)
			• “… having one recruiting drive at the time. So, it’s like, you know, singularly
			• attack, we focus on this one this period of time and there’s a sort of a cut off for it” (P2)
			• the more studies that there are in-in the pack that you have to offer, the more- the more difficult it is to-to sort of push forward (P6)
	Study design and focus	Name of the study	• “…because it’s got depression in the title and um I think um you it just seems a little bit more explanation um by inviting them to take part I’m not suggesting that they are depressed…” (P1)
			• “I prefer not to call it a depression diagnosis study explicitly when I’m talking about it with families but that wouldn’t be the first thing I would say I would introduce it to you know a study that’s looking into young people’s experiences of you know moods and emotions and I would want to sort of say something a little bit broader um but that’s more perhaps a personal approach.” (P3)
			• “…sometimes you won’t even think of it if you’ve got a patient who clearly isn’t depressed at all is coping extremely well it doesn’t even cross your mind to enter them into something called the depression study so I think the name of the study is wrong it should be called you know mood in chronic fatigue or something to help people think it’s for everyone, we’re just as interested in people who don’t have depression as people who do. So the name was wrong” (P5)
		The focus of the study - Patient perceptions of a mental health study	• “…can meet some resistance sometimes and one of-I had one mum who said I think her child was um her daughter was thirteen and she didn’t want her to take part because she didn’t want her-she felt she was too young and that being asked questions about her mood would kind of put ideas into her head, that was what she felt.” (P1)
			• “because it is more objectively more obviously about the mental health side of things I have found it to be a different experience recruiting to this” (P3)
			• “…when I have younger patients the opening sheet which you show them that says we’re going to talk about sex and smoking or drinking whatever some parents balk at and they think oh that’s too intrusive I don’t want to do that study.” (P5)
		- Healthcare professionals’ concerns about it being a mental health study	• “I’m here for your physical health; you know obviously please do tell me about any…mental health um concerns, … but ultimately …the sessions with me aren’t…mental health sessions…I’ve been in physical health since I qualified … so I don’t feel as though I am up-to-date you know mental health” (P1)
			• “if a young person is then having a research interview with the researcher and something comes up along the lines of risk that you know that then needs to be managed there’s always things in place that when you add research to the package to the interactions with the service it opens up other bits that need managing or organising or accounting for.” (P3)
		It being an observational study	• “I think the difference of priority is – is that, um with MAGENTA you’re recruiting into a treatment trial--an intervention. With the depression study you’re recruiting into a semi-structured interview, the outcome of which may or may not benefit the--young person.” (P2)
			• “…it doesn’t lead to additional help or additional resource or anything, it is simply, um a snapshot of the people coming through which is very valuable …but look what’s – what’s the benefit for the client?” (P2)
What helps to support recruitment	Study familiarisation		• “I think if it-if it feels like something distant…that can make it more difficult I think for me to speak with kind of conviction…” (P1)
			• “…it’s about understanding why the research is useful, but I need to understand the content, otherwise I-I wouldn’t feel confident in consenting actually” (P2)
	Tools to support recruitment		• “I really, really find it helpful that all the research packs are done so you know the information sheets are printed and often massive time saver that they are kind of bundled together and ready for a clinician to give.” (P3)
			• “having the recruitment form in the notes when you see a patient so it’s there already and you don’t have to remember to carry it with you” (P6)
	Use of Reminders		• “email updates of where recruitment lies or how it’s growing and essentially reminders, keep this study on your radar we’re still recruiting that’s been hugely helpful. Hugely, hugely helpful.” (P3)
			• “reminders…that was probably helpful just to keep it in mind” (P2)
			• “[emails are] kinda good but it doesn’t jump to the top of my inbox, it doesn’t get flagged” (P2)
			• “having the updates in the um team meetings is useful too…in fact that’s probably the key- the heart of it really” (P6)
			• “..the checking afterwards from the research team so for example if I’ve seen somebody and not recruited them and I haven’t um given the information yet as why they were not recruited then that email coming that says oh can you just let me know what the reason was and then I can make sure that I’ve done that so that data doesn’t get lost in the ether” (P3)
			• “I get the email saying you’ve got this patient coming up could you consider them for the study. Those sorts of things are just so helpful because there’s so many balls to juggle and it’s just that sort of prompt” (P4)
	Being able to recruit at multiple points, not just initial assessment		• “…multiple points of recruitment so sometimes I think someone might in time want to enter the study but it’s not appropriate in the initial consultation so being able to contact them at a later date I think would be equally valid and important actually.” (P5)
			• “being able to defer it to the second appointment” (P6)
	Value of case specific information (outcome of the study)		• “… if I was seeing follow ups and I was seeing the results of the mood interviews and digested them before the follow up that would be really helpful. So when it goes into great detail and looks at different levels of anxiety and harm and obsessive behaviour and things like that I think that’s really useful for the future.” (P5)
			• “[substance abuse disclosures] comes as a bit of a surprise from some of the interviews…that would be my learning point from what we’ve been doing so far”(P6)

P1, P2, etc. denote nominal participant identification numbers

### The value of research

Participants expressed their interest in and **commitment to research** (subtheme 1); “*it’s very positive because we’re making change to help the whole population*” (P4). Working in a research active team was viewed as part of the role; “*my job is to make sure that if there is anybody eligible that I recognise that and I offer them the opportunity to take part*” (P6). Research activity was even a reason why participants had chosen to work in this particular team, who found it “*really exciting…to be involved-to have that link*” (P1). Participants highlighted the **importance of developing the evidence base through research** (subtheme 2) as a way to “*answer unanswered questions*” (P5), which gives them “*added confidence*” (P1) when conveying information to patients seen in the service. However, although participants valued research, they did not see it as their primary focus or role (subtheme 4), with their priority being clinical care; *“[research] is not the emphasis of my assessment…because I’m here for clinical end of things*” (P2).

### Barriers to recruitment

A number of barriers to recruitment were highlighted by participants, which clustered into 3 subthemes.

**The clinical context of the recruitment activity** (subtheme 1), which in this study was the initial clinical assessment, raised barriers. The first barrier (subtheme 1a) was “*limited time to get all the information out*” (P4), which meant that participants had to prioritise what to spend time on and research studies might not be prioritised. Another contextual barrier was more complex, demanding and emotional initial assessments (Subtheme 1b), where participants may not discuss research “*if the patient is struggling to take on information about their diagnosis let alone an additional bit of work around research*” (P4). Participants recognised that this meant that some patients were more likely to be recruited than others, which results in “*bias”* (P6), despite the participants recognising how important it is to offer all patients the option of participating in research studies.

The **wider research context** (subtheme 2) also presented barriers, including other studies being conducted concurrently in the same service which created research recruitment burden, “*research overload*” (P5). It also created confusion as “*there were around four or five research projects, seem to be, seemingly on the go at any one time…and that is confusing*” (P2).

**Factors specific to the study** including study design and focus were also highlighted as barriers (subtheme 3). This included the name of the study (subtheme 3a), which participants perceived as being off-putting to potential participants (the ‘Depression Diagnosis Study’, although as a prevalence study, the aim was to recruit a clinical cohort of patients with CFS/ME, not just those with co-morbid depression). This meant that more explanation was needed to clarify that “*by inviting them to take part I’m not suggesting that they are depressed…*” (P1). The name of the study also meant that it didn’t occur to participants to recruit participants for whom depression did not seem to be an issue, *“…if you’ve got a patient who clearly isn’t depressed at all is coping extremely well it doesn’t even cross your mind to enter them into something called the depression study*” (P5).

The focus of the study on mental health (subtheme 3b) raised further barriers, the first of which was patient and family’s perceptions of mental health in CFS/ME which “*can meet some resistance*” (P1) and the sensitivity of the topics discussed in the research interviews (e.g. sex, substance use) which is “*too intrusive*” (P5).The second barrier raised by the focus of the study was the participants’ own training, and their perceived lack of qualifications and/or resources to address mental health problems, in the context of a physical health setting where staff “*don’t feel as though I am up-to-date [with] mental health*” (P1). The nature of the study as an observational study rather than an intervention study (subtheme 3c) was also seen as a barrier, as participation was not necessarily viewed as generating a beneficial outcome for the specific patient making it a “*harder sell”* (P6), despite the recognition of the wider value of research.

### Recommendations for promoting recruitment

Participants shared their ideas about how to support recruitment. These included ensuring that the gatekeepers understand that content of the study as part of **study familiarisation** (subtheme 1); “…*understanding why the research is useful…I need to understand the content, otherwise I-I wouldn’t feel confident in consenting actually*” (P2). **Tools to support recruitment** included making it as easy as possible for the gatekeeping healthcare professional to recruit including enclosing printed copies of the study materials in each set of patient notes (subtheme 2); “*I really, really find it helpful that all the research packs are done so you know the information sheets are printed and ready for a clinician to give”* (P3). **Use of reminders** which are both generic and linked to specific potentially eligible patients was suggested by some participants (subtheme 3); “*reminders, keep this study on your radar, we’re still recruiting that’s been hugely helpful*” (P3), although other participants found these less helpful as “*I get the emails but I get such a load of emails*” (P2) and the reminders may even be “*irritating*” (P6), especially when faced with multiple clinical pressures. More **flexibility in the timing of the recruitment activity** so that patients can be recruited at the first follow-up appointment if time pressures or the emotional context of the initial assessment precludes recruitment as “*someone might in time want to enter the study but it’s not appropriate in the initial consultation*” (P5) was suggested (subtheme 4). Highlighting the positive outcomes of the study and its **value on a case-by-case basis** (subtheme 5), with participants valuing the feedback from individual interviews and identifying “*learning points”* (P6).

## Discussion

Multidisciplinary healthcare professionals who were actively acting as gatekeepers for the initial stage of recruitment to an observational study of mental health in adolescents with CFS/ME all valued research, but perceived there to be a number of contextual and study specific factors which acted as barriers to recruitment. The clinical context of the recruitment activity, including a lack of time, and the emotionally demanding nature of the initial clinical assessment, as well as the wider research context of competing studies were apparent barriers. The name of the specific study, which was experienced as off-putting and misleading to patients, the sensitive nature of the questions asked in the study, participants’ own lack of training and resources to manage emergent mental health problems, and the lack of a direct beneficial outcome for the patients, were also cited as barriers. Recruitment activities could be supported by study familiarisation, regular reminders, greater flexibility about the point of recruitment, promoting the value of the case specific information gained from participating in the study and thinking carefully about the name of the study.

The clinical context, in this study, was perceived to be a barrier to recruitment; participants talked about the pressures of time within the initial clinical assessment as the recruitment point. Contextual barriers have been reported in previous studies [13] which have recommended making it as easy as possible for clinical gatekeepers to assess for eligibility and seek consent to contact from the research team [14, 15]. Providing easily accessible study information such as printed study recruitment packs is critically important. In interventional studies, recruiting and potentially randomising participants prior to their having received any interventions is an important part of study design; therefore, the initial clinical assessment is a key recruitment point. In an observational study, determining a consistent point of recruitment is important, but it may be worth having some flexibility (for example, to recruit at the first follow-up) or recruiting at a subsequent point in a patient’s journey through a specialist service where this is possible within the requirements of the study design. However, the gatekeeping activities within this study did not differ from that of previous studies within the same clinical context, although the rates of assessment for eligibility did. Therefore, it may be that factors specific to this study hampered gatekeeping activities beyond that which would be accounted for by the clinical context.

Recruitment problems in paediatric studies specifically are compounded by added complexity of requiring parental consent and requiring time out of school for children and potentially out of work for parents; across 181 Principal Investigators on paediatric trials at the Boston Children’s Hospital, 46% of studies were reported to have experienced delays in recruitment [6]. Our finding that gatekeepers may assume that patients and families may find it too overwhelming to be invited to participate in research at the end of an emotional session might be the result of paternalism, which has also been found in previous research [8, 13] and it may be that further education of healthcare professionals about the potential benefits to patients of participating in research is indicated. However, further work with patients and their families would be important to ensure that research is introduced in the most sensitive manner possible, and at the most appropriate time, and it is possible that there may be risks associated with participation in psychiatric studies, such as a potential worsening of mental state as a result of the research procedures which may potentially elicit distress or discomfort [9].

Furthermore, the wider research context can create barriers to recruitment. Previous studies also found that have numerous competing studies recruiting in parallel within one clinical location can serve as a barrier to recruitment. Fenlon et al. [1] found that clinical staff are reluctant to recruit to more than 1 study at a time; in the current study, the patient eligibility criteria for the studies open to recruitment meant that each patient could only be recruited to 1 study, although they were potentially eligible for more than 1 study. Studies have also found that clinicians feel bombarded with too much research [13]. Therefore, carefully considering the timeframes of recruitment for all the research activities within a clinical setting, and having a clinical team member who leads on overseeing research could enable better management of research demands arising from multiple studies recruiting in parallel. Having a clinical team member who champions research has also been recommended elsewhere [1, 14], with the added benefit that these individuals can be involved from design stage [13]. Prompts for the gatekeepers to remind them about the research, both generic and patient-specific, can be helpful.

Study design also seems to be very important as the nature of the research may also affect the views of gatekeepers. The current study was an observational study, and participants commented that they did not perceive there to be a direct benefit to patients of participating in the study, which discouraged them from undertaking study related gatekeeping activities. It may be that the research team could have further highlighted potential benefits of participating to the clinical gatekeepers, particularly at study initiation stage. Prior studies have found that drug studies are more likely to be given priority over epidemiological studies [1], although clinicians recruiting to research studies have also reported that clinical trials are harder to recruit to as they demand greater commitment from participants, both with regard to time and risk [2].

Another barrier in the current study was the gatekeepers’ perceptions of a lack of training and resources to deal with any mental health issues that emerged as an outcome from the research assessments. This may have led to selection bias, with gatekeepers being less likely to introduce the study to patients who were more distressed.

Importantly, the current study found that the name of the study is important to consider, particularly in the context of stigmatised conditions such as mental health, where patients may be reluctant to participate by association [8]. Whilst the study name needs to be appealing, it should not be misleading. Involving key stakeholders, including patients and clinical gatekeepers, at design stage to generate a meaningful and appropriate study name could prevent the name from subsequently posing a barrier to recruitment.

Arguably, due to these barriers, in this study, patients were denied access to taking part in research. All potential participants should be given the option of participating in studies. The NHS constitution commits to informing all patients of research studies that they are eligible to participate in [16], consistent with the ethos of ‘No decision about me without me’ [17]. Participating in research may be therapeutic and empowering, giving patients an additional opportunity to talk about their experiences [9, 18].

### Strengths and limitations

We had good rates of study update, with 6 of 7 potentially eligible healthcare professionals participating in the interviews. However, this was a small sample in the context of one specialist paediatric service within the National Health Service, which limits the generalisability of these findings. Nevertheless, many of the themes are widely applicable and are consistent with those in other paediatric and mental health studies. Some interviews were conducted remotely and others in person, but we do not think that this unduly influenced the respondents’ narratives. The topic of recruitment to paediatric studies would benefit from further investigations in a variety of settings, and from different stakeholder perspectives.

## Conclusion

Gatekeeping professionals generally valued research, but highlighted a number of barriers to recruiting, related to both the wider research and clinical contexts, and to the design of a study specifically. The current study highlights the need to carefully consider, at design stage, how to overcome potential barriers to recruitment where it is possible to do so (see Table 2). Gatekeepers should be actively involved at this stage to ensure that the study is set up in such a way to best enable recruitment activities within the clinical setting. It is also worth spending time educating busy clinical gatekeepers about the study to ensure that they understand its relevance and importance.

**Table 2:** Recommendations for how to support recruitment

Study design:	
o Maximise flexibility in the point of recruitment, having multiple potential points of recruitment where possible	
o Consider how many studies are recruiting from the same setting in parallel and aim to limit this	
o Include gatekeepers at design stage to ensure that all foreseeable problems are addressed as early as possible	
o Carefully consider the name of the study, including consulting with all relevant stakeholders about this	
Study information:	
o Make it easy for gatekeepers to access study materials during clinical sessions	
o Use generic and patient specific reminders	
Interface with the gatekeepers:	
o Ensure that the gatekeepers understand that content of the study	
o Highlight the positive outcomes of the study and its value	
o Educate gatekeepers about the potential benefits to patients of participating in research	
o Engage a member of the clinical team to champion research	

## Abbreviations

CFS/ME: Chronic Fatigue Syndrome/Myalgic Encephalomyelitis
NHS: National Health Service
